# Assessment of TTX-s and TTX-r Action Potential Conduction along Neurites of NGF and GDNF Cultured Porcine DRG Somata

**DOI:** 10.1371/journal.pone.0139107

**Published:** 2015-09-25

**Authors:** Robin Jonas, Andreas Klusch, Martin Schmelz, Marlen Petersen, Richard W. Carr

**Affiliations:** Department of Anesthesiology, Medical Faculty Mannheim, University of Heidelberg, Mannheim, Germany; Monash University, AUSTRALIA

## Abstract

Nine isoforms of voltage-gated sodium channels (NaV) have been characterized and in excitable tissues they are responsible for the initiation and conduction of action potentials. For primary afferent neurons residing in dorsal root ganglia (DRG), individual neurons may express multiple NaV isoforms extending the neuron’s functional capabilities. Since expression of NaV isoforms can be differentially regulated by neurotrophic factors we have examined the functional consequences of exposure to either nerve growth factor (NGF) or glial cell line-derived neurotrophic factor (GDNF) on action potential conduction in outgrowing cultured porcine neurites of DRG neurons. Calcium signals were recorded using the exogenous intensity based calcium indicator Fluo-8®, AM. In 94 neurons, calcium signals were conducted along neurites in response to electrical stimulation of the soma. At an image acquisition rate of 25 Hz it was possible to discern calcium transients in response to individual electrical stimuli. The peak amplitude of electrically-evoked calcium signals was limited by the ability of the neuron to follow the stimulus frequency. The stimulus frequency required to evoke a half-maximal calcium response was approximately 3 Hz at room temperature. In 13 of 14 (93%) NGF-responsive neurites, TTX-r NaV isoforms alone were sufficient to support propagated signals. In contrast, calcium signals mediated by TTX-r NaVs were evident in only 4 of 11 (36%) neurites from somata cultured in GDNF. This establishes a basis for assessing action potential signaling using calcium imaging techniques in individual cultured neurites and suggests that, in the pig, afferent nociceptor classes relying on the functional properties of TTX-r NaV isoforms, such as cold-nociceptors, most probably derive from NGF-responsive DRG neurons.

## Introduction

Voltage gated sodium channels (NaV) underlie action potential initiation and conduction in excitable tissues. Nine NaV isoforms have been functionally expressed and have been nominally categorized according to their sensitivity to the bacterial guanidium toxin tetrodotoxin (TTX). Six NaV isoforms are sensitive to TTX (TTX-s) at nanomolar concentrations (NaV 1.1–4 and NaV1.6–7) and three isoforms (NaV1.5, NaV1.8 and NaV1.9) are resistant to TTX (TTX-r) into the mM range owing to a single residue mutation in the outer pore loop of domain I [1]. Small diameter C-type neurons with unmyelinated axons express different admixtures of the TTX-s isoform NaV1.7, and possibly NaV1.6, together with the TTX-r isoforms NaV1.8 and NaV1.9 [2] both of which are associated primarily with nociceptive neurons [3]. Individual DRG neurons can express multiple NaV isoforms, a feature potentially allowing different NaV isoforms to subserve specific functions in different neuronal compartments. For example, the peripheral nerve terminals of nociceptors are able to generate action potentials during strong cooling by virtue of NaV1.8 resisting temperature dependent inactivation [4]. In the somata of DRG nociceptors, a large fraction of inward current during the somal action potential is TTX-r [5] specifically mediated by NaV1.8 [6].

In DRG neurons, the expression level of NaV1.8 is correlated with increased action potential overshoot and longer duration action potentials [7] where it can support high frequency repetitive firing [6]. Along the axons of DRG neurons, the NaV isoforms contributing to action potential conduction differ according to myelination. NaV1.6 underlies the nodal sodium current in myelinated axons [8] while unmyelinated axons may express NaV1.7 and possibly NaV1.6 [9] together with the TTX-r isoforms NaV1.8 and NaV1.9 [10]. Using TTX as a probe, the balance of evidence suggests that, for mammalian C-fibers TTX-s NaV isoforms are primarily responsible for action potential conduction [4,11,12,13,14,15,16]. Although TTX-r NaV isoforms can support axonal conduction over short lengths and at low temperatures in a small percentage of unmyelinated axons [17,18], the use of isoform specific μ-conotoxins implies that NaV1.7 is the primary mediator of conduction in unmyelinated axons [19]. Consistent with the primary role of TTX-s NaV isoforms in axonal conduction, action potential conduction in vagal unmyelinated axons is abrogated by shRNA knockdown of NaV1.7 in guinea-pigs [20]. In the central terminals of unmyelinated primary afferents TTX-s NaV isoforms are also essential for fast synaptic transmission in the dorsal horn [21].

NaV expression in DRG neurons can be differentially regulated by neurotrophic factors. Following axotomy, DRG neurons downregulate expression of TTX-r NaV isoforms and upregulate rapidly repriming TTX-s NaV1.3 [22]. For NaV1.8 this reduction can be rescued by nerve growth factor (NGF) [23], while glial cell line-derived neurotrophic factor (GDNF) partially normalizes expression levels of both NaV1.8 and NaV1.9 [24]. In vivo, neuronal DRG expression of NaV 1.8 is upregulated in mice in response to the inflammatory stimulus complete Freund’s adjuvant [25]. Neurotrophic factors are able to exert different effects on NaV expression in DRG sensory neurons because individual DRG neurons express different constellations of neurotrophic receptors. In rodents, two major classes of DRG nociceptors can be delineated by their specific expression of neurotrophic receptors. One population responds to NGF throughout development and in adulthood by expressing both the high-affinity tropomyosin kinase (trkA) as well as the low-affinity p75 neurotrophin receptor. The other population downregulates TrkA expression postnatally and becomes sensitive to GDNF via expression of the common GDNF family receptor cRet and a specific co-receptor from the GDNF family receptor alpha (GFRα) [26,27]. These differences in neurotrophic receptor expression correlate with histochemical markers and functional properties. In rodents, DRG neurons expressing receptors for NGF have higher TTX-r sodium current densities, longer duration action potentials and express the neuropeptide CGRP, while GDNF-receptor expressing DRG neurons are labeled by the isolectin IB4 [28,29]. The current study aimed to explore further functional differences between NGF and GDNF responsive DRG neurons by examining the role of NaV isoforms in axonal conduction along cultured neurites. A porcine model was used because of the similarity of functional types of DRG nociceptors in the pig and people [30].

## Methods

### Animal preparation

Ethical approval for experimental procedures was issued by the Ethics committee of the regional government (Karlsruhe, Baden-Wuerttemberg, Germany).

Dorsal root ganglia (DRG) were removed post-mortem from male piglets (*Sus scrofa domesticus*) ranging in age from P9 to P14. Piglets were initially sedated with intramuscular azaperone (Janssen-Cilag GmbH Neuss, Germany; 28 mg / kg) and ketamine (Essex Pharma GmbH, Munich, Germany; 70 mg / kg) and subsequently killed with a lethal dose of intracardial pentobarbital (20 mg / kg). The spine was removed, cleaned and stored in cold PBS (Sigma-Aldrich, Seelze, Germany). Following mid-sagittal section, DRG were removed from all levels of the spinal cord and placed in DMEM (Sigma-Aldrich, Seelze, Germany).

### Cell culture

Isolation and culture of DRG neurons was similar to previously described procedures [31,32]. Briefly, DRGs were freed mechanically from connective tissue and incubated at 37°C for 110 min in DMEM containing Gentamicin and collagenase (Invitrogen, Life Technologies, Schwerte, Germany) during which half of the medium was replaced with fresh medium twice. Ganglia were rinsed twice in PBS devoid of Ca^2+^ and Mg^2+^ and incubated for 8 min at 37°C in trypsin (Sigma-Aldrich, Seelze, Germany). Ganglia were placed in a mixture of DMEM and Ham’s F-12 (Gibco, Life Technologies, Schwerte, Germany) and triturated with a fire-polished siliconized Pasteur pipette to isolate somata. Cells were subsequently transferred to 10% Percoll solution and centrifuged (740 RZB) to remove connective tissue. The resulting pellet was washed twice in DMEM and centrifuged (170 RZB).

Cells were cultured in the central compartment of a Campenot chamber [33] in Ham’s F12 medium supplemented with 10% heat-inactivated horse serum (Gibco, Life Technologies, Schwerte, Germany), 2 mM L-glutamine, 100 U / ml penicillin and 10 μg / ml streptomycin. Medium was supplemented with either rhß-NGF (50 ng / ml Calbiochem, Schwalbach, Germany) or rh-GDNF (50 ng / ml R&D System, Minneapolis, USA) and anti-NGF (2 μl / ml Sigma-Aldrich, Seelze, Germany). NGF cultured somata only extend neurites into the lateral compartment of the Campenot chamber in the presence of NGF in both the lateral and central compartments [34,35]. In contrast, somata cultured with GDNF in the central compartment extend neurites into the lateral compartment in the absence of any neurotrophins in the lateral compartment. To study different neuronal populations we cultured DRG neurons either in NGF (central compartment 50 ng / ml and lateral compartment 100 ng / ml) or GDNF (only central GDNF 50 ng / ml and anti-NGF 2 ng / ml). For simplicity, neurites of DRG somata cultured in NGF or GDNF are termed “NGF neurites” or “GDNF neurites” in the manuscript. Cells were kept in culture at 37°C in a 5% CO_2_ humidified atmosphere and half of the medium was replaced every 2–3 days. Experiments were performed after 4–9 days in culture.

### Calcium imaging setup

In preparation for calcium imaging, the culture medium was removed from all 3 compartments of the Campenot chamber and neurons were washed three times with imaging buffer (in mM: 140 NaCl, 2 KCl, 2 CaCl_2_ x 6 H_2_O, 1 MgCl_2_ x 6 H_2_O, 20 d-glucose, 10 HEPES, pH 7.4). After washing, the intensity based calcium indicator Fluo-8®, AM (AAT Bioquest, Sunnyvale, CA, USA), diluted in imaging buffer to a concentration of 2 μM, was added into all compartments. Cells were incubated in fluorescent dye for 30 min at room temperature before being washed three times with imaging buffer and left to stand for a further 20 min. This incubation and rinse procedure was performed in a darkened room. Fluorescence and brightfield images were acquired using a back-illuminated 512 * 512 pixel cooled EMCCD camera (Evolve 512, Photometrics, Tucson, AZ, USA). The camera was connected to the side port of an inverted microscope (Axiovert 200, Zeiss, Jena, Germany). Bright-field illumination was provided by the microscope’s halogen lamp. A 465 nm LED (Prior Scientific, MA, USA) and a filter set (excitation BP 450–490 nm, dichroic = 510 nm, emission = 515 nm LP, Chroma Technologies) were used for excitation of Fluo-8®, AM. Fluorescence images were acquired using μManager [36] software with electrical stimulation and image acquisition synchronized via an Arduino Duemilanove (Watterott electronic, Leinefelde, Germany). Fluorescence image time sequences were recorded at 1 Hz except during the 4 s period beginning with electrical stimulation during which images were acquired at either 5 Hz or 25 Hz.

### Experimental protocols

#### Control electrical stimulation

**Fig 1:**
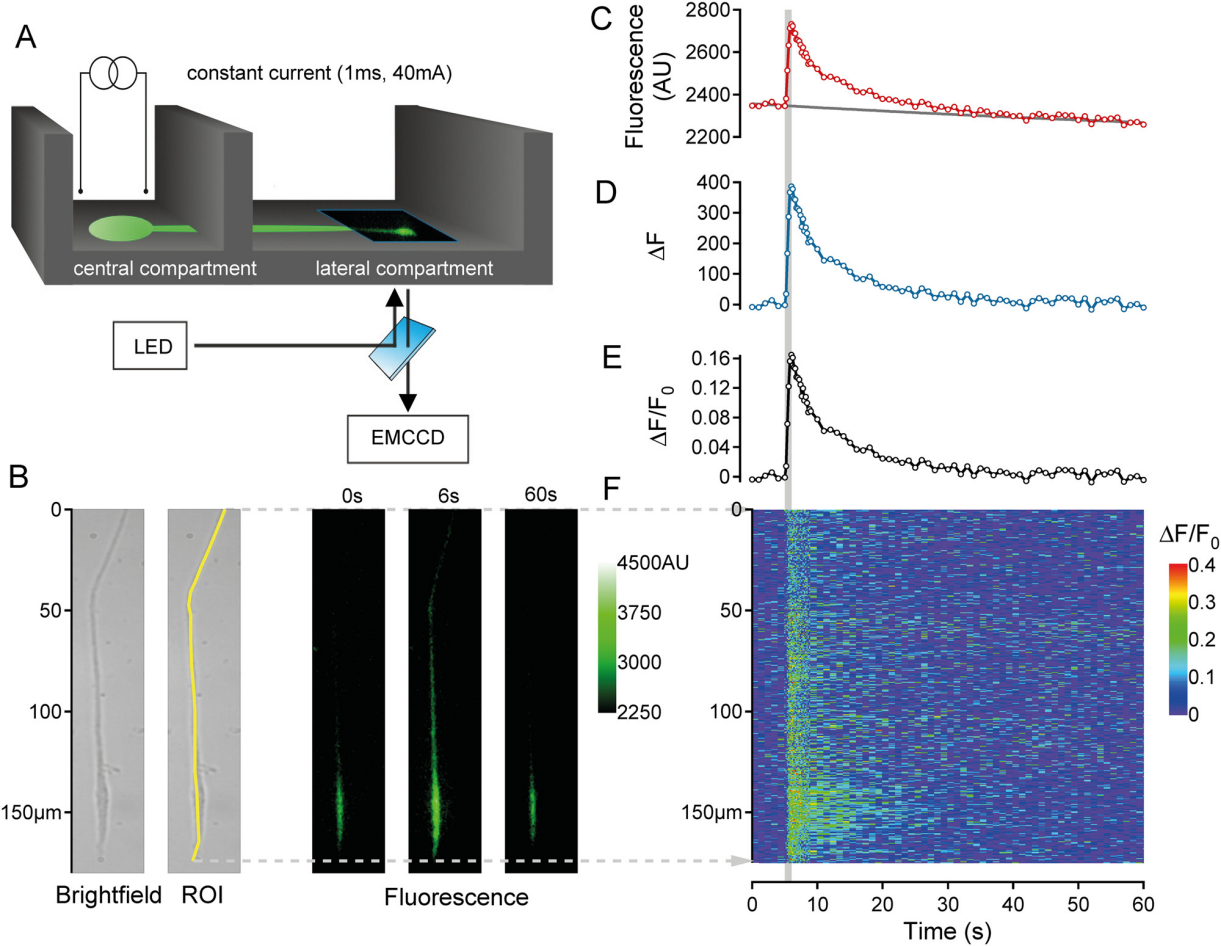
Recording and analysis of calcium signals in outgrowing neurites. (A) Schematic illustrating DRG neurons (green) cultured in the central compartment of a Campenot chamber that extended neurites into the lateral compartment. Fluorescent images were acquired from the lateral compartment. Constant current field stimulation (1s, 40mA, 20Hz) was applied in the central compartment. (B) Neurites were initially identified under brightfield illumination (B, left) and calcium (Fluo-8®, AM) fluorescence images (B, right) were acquired sequentially at 1Hz for 60s, with an increase in acquisition rate to 5Hz during the 5-10s period. A region of interest (ROI) was selected from the brightfield image (B, left) and used to determine average fluorescence (AU) within the ROI (C, red markers) for each image across time. A single exponential fit was used to determine baseline fluorescence (F_0_; C, solid grey line). The change in fluorescence (ΔF = F-F_0_; D, blue markers) was determined as the difference between raw (C, red markers) and fitted baseline fluorescence values (C, solid grey line). This difference signal was subsequently divided by the baseline fluorescence (C, solid grey line) to yield a fluorescence ratio (ΔF/F_0_) (E, black markers). In addition to average fluorescence values (C-E) the spatio-temporal distribution of fluorescence (panel F, color-coded) was determined by calculating the fluorescence ratio (ΔF/F_0_) for each individual pixel along the ROI (F, y-axis in μm) as a function of time, i.e. each image during the acquisition period (F, Time axis).

Neurites were first identified under bright-field illumination in the two lateral compartments of the chamber (Fig 1A). Initially, neuronal vitality was determined by monitoring the calcium response to electrical stimulation (Fig 1B). Constant current field stimulation (20 pulses at 20 Hz, 40 mA, 1 ms) was applied in the central compartment (Digitimer DS7A, Letchworth Garden City, UK). Neurites that did not respond with an increase in calcium to electrical stimulation were not further studied.

#### NaV blockade

After the initial control stimulation the protocol comprised 4 sequential 10 minute periods in which the neurite was perfused with tetrodotoxin (TTX, 500 nM) followed by washout of TTX and then wash-in of lidocaine (1 mM) and finally washout of lidocaine. Test substances were added to the perfusate for the lateral compartment. At the end of the perfusion period, the calcium response to electrical stimulation was determined. Each substance was washed in/out at a flow rate of 3 ml / min (Minipuls®3, Gilson, Middleton, WI, USA).

#### Calcium replacement experiments

Two manipulations were used to examine the source of the increase in intracellular Ca^2+^ seen in response to electrical stimulation. To examine the contribution of extracellular calcium, either Ca^2+^ (2 mM) in the imaging buffer was replaced with an equimolar concentration of Mg^2+^ (2 mM) or EDTA (3 mM) was added to the extracellular solution. EDTA (3mM) with Ca^2+^ (2mM) would be expected to result in a solution containing approximately 0.07 mM free calcium (http://www.stanford.edu/~cpatton/webmaxcS.htm).

#### Frequency dependence of electrically-evoked calcium fluorescence transients

To examine peak fluorescence intensity as a function of electrical stimulation rate, responses to 1 s bouts of electrical stimuli comprising 1, 5, 10, 20, 50 or 100 pulses were recorded at intervals of not less than 120 s. Subsequent to electrical stimulation, ionomycin (10 μM) was applied to the lateral compartment to determine the maximum calcium fluorescence intensity. Image acquisition rate before and during ionomycin exposure was 0.1 Hz.

### Data Analysis

Image analysis was performed using ImageJ-software (NIH). Regions of interest (ROI) were delineated by hand by drawing a line along the entire length of the visible neurite as seen in the bright-field image (Fig 1B). The time profile of intensity for each pixel along this linear ROI was determined from each fluorescence image using the Reslice function in ImageJ. The resulting data array was processed using custom written routines in Igor Pro (WaveMetrics, Lake Oswego, OR, USA).

Fluorescence intensity (F) signals were averaged across all pixels of the ROI at each time point. The resulting time series was corrected for bleaching in the following manner. A single exponential function was determined using the initial 5 control values and the last 20 values of the fluorescence time series (Fig 1C). The difference between raw and fitted fluorescence values (Fig 1D) was divided by the fluorescence fit value at each time point to yield the fluorescence ratio (ΔF/F_0_, Fig 1E). To quantify calcium responses to electrical stimulation the average before stimulation, maximum positive peak and area under the curve (AUC) were calculated from ΔF/F_0_ values. In addition, spatial changes in fluorescence intensity were calculated by determining ΔF/F_0_ as detailed above for each individual pixel within the ROI across time (Fig 1F).

To evaluate whether or not a calcium signal was evoked in response to electrical stimulation the mean and standard deviation (SD) of ΔF/F_0_ was calculated for the 5 values during the baseline period for each neurite. Positive calcium responses were defined as those showing at least 2 consecutive values of ΔF/F_0_ during the stimulation period that were above the baseline mean + 2 SD. All fluorescence images shown in the figures were modified using a 3x3 median filter. For the regression of calcium response amplitude and electrical pulse number an exponential fitting function was used.

### Chemicals

All chemicals were obtained from commercial sources. Tetrodotoxin citrate (Tocris, UK) and lidocaine hydrochloride (Sigma-Aldrich, Seelze, Germany) were made up in stock solutions of PBS and distilled water respectively. Stock solutions of ionomycin (10 μM, Tocris, Bristol, UK) were made up in dimethylsulfoxide (DMSO). Stock solutions were aliquoted and stored frozen before being diluted to the desired concentration in imaging buffer on the day of the experiment.

### Statistics

Statistical tests were performed using PASW Statistics 18 (SPSS Inc., Chicago, IL, USA). The effects of lidocaine and TTX on ΔF/F_0_ were examined using two-way repeated measures ANOVA. Student’s t-test for unpaired samples was used to compare fluorescence intensity, AUC and decay of electrically evoked calcium signals between NGF and GDNF cultured neurons. Pearson's chi-squared test was used to compare the incidence of complete conduction block in the presence of TTX. Group data are presented as mean ± standard error of the mean. The level of statistical significance is indicated in the figures as either * for *P* < 0.05 or ** for *P* < 0.01.

## Results

Fluorescent calcium signals were recorded from 94 individual neurites from 9 piglets using the intensity based fluorescent dye Fluo-8®, AM. Neuronal somata were cultured either in NGF (n = 49) or GDNF (n = 45). The multiple compartment configuration of the culture chamber allowed us to electrically stimulate in the central compartment and monitor calcium signals of the outgrowing neurites in the lateral compartment (Fig 1). For both, GDNF and NGF-cultured somata it was possible to record fluorescent calcium signals in neurites in the lateral compartment in response to electrical stimulation delivered to the central compartment. These electrically-evoked calcium signals in neurites could be blocked by the broad spectrum sodium channel (NaV) blocker lidocaine (1 mM) applied singly to the central compartment, whereby any electrical stimulus spread from the central into the lateral compartment was insufficient to activate the neurites (data not shown). This is consistent with calcium signals resulting from action potential propagation along neurites from the central compartment into the lateral compartment. Electrotonic spread of depolarization along neurites from the central compartment into the lateral compartment is rendered unlikely because the physical barrier separating the central and lateral compartments was 1mm wide (Fig 1A). A 1mm barrier plus the remaining length of neurite in the lateral compartment is somewhat longer than the estimated length constant of neurites of approximately 160–350 μm, assuming a diameter of 1–5 μm (specific resistivity of the membrane of 1000 ohm.cm^2^ and the axoplasm of 100 ohm.cm [37]).

Under control conditions, electrically-evoked calcium signals in NGF- and GDNF neurites did not differ significantly with respect to fluorescence intensity (n = 94, p = 0.495, unpaired Student’s t-test), time to peak (n = 94, p = 0.232, unpaired Student’s t-test) and AUC of the fluorescence intensity change over the 30 s period following electrical stimulation (AUC, n = 94, p = 0.440, unpaired Student’s t-test). There were no correlations between the age of the piglet and peak changes in fluorescence (n = 94, r = -0.18) or AUC (n = 94, r = -0.1). Similarly, the number of days in culture did not correlate with the peak change in fluorescence (n = 94, r = -0.01) nor AUC (n = 94, r = -0.07).

### Role of extracellular Ca^2+^ in electrically-evoked calcium responses in neurites

**Fig 2:**
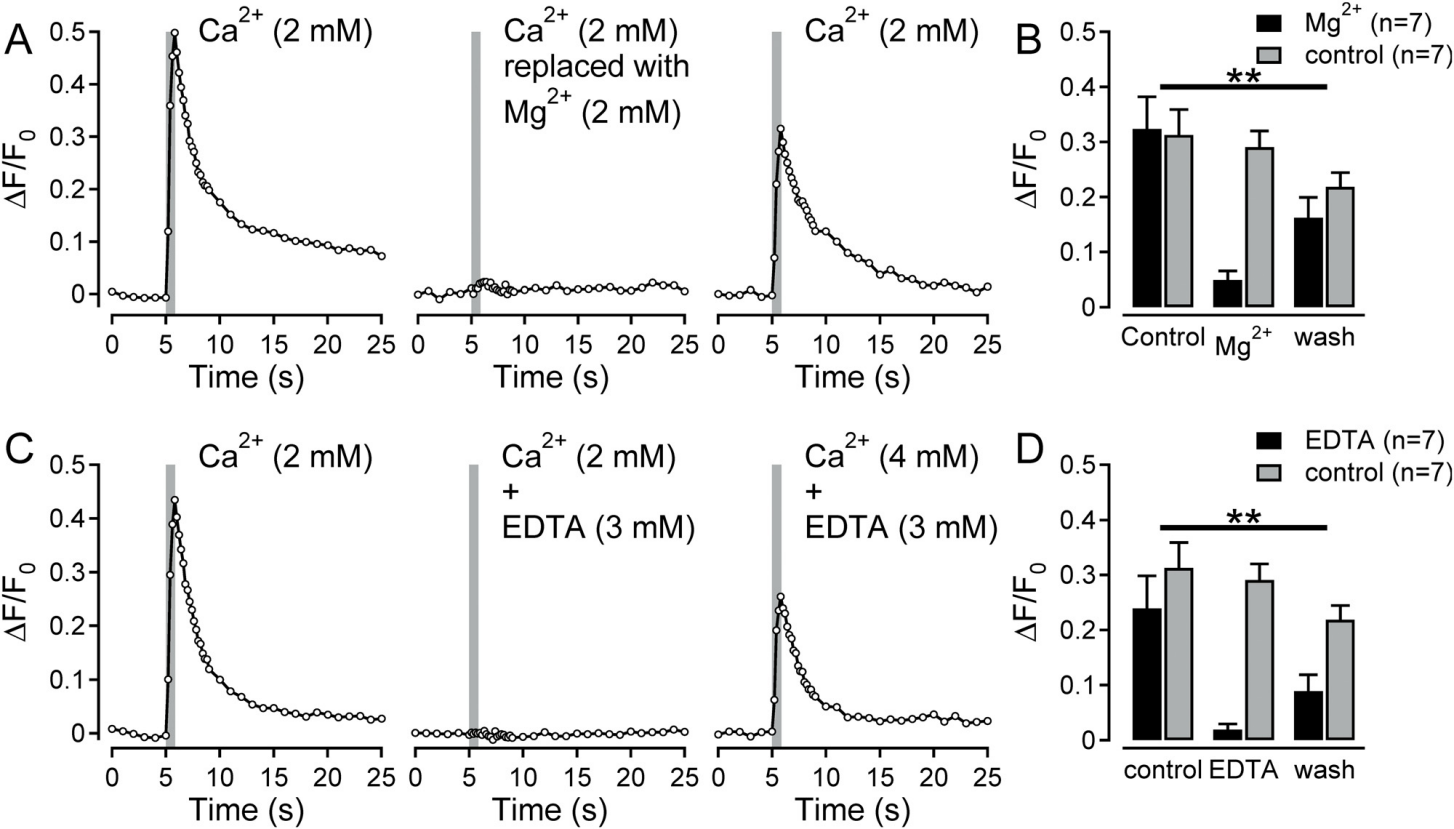
Role of extracellular Ca^2+^ in electrically-evoked calcium responses in neurites. (A) Fluorescent calcium responses to three bouts of electrical stimulation in a single neurite under control conditions (left panel), following exchange of extracellular Ca^2+^ for equimolar Mg^2+^ (centre panel) and after wash with re-establishment of the extracellular Ca^2+^ concentration (right panel). (B) Pooled data for 14 neurite recordings comparing repeated electrical stimulation (grey bars, control) with the intervention of replacing extracellular Ca^2+^ with Mg^2+^ (filled bars). Calcium responses during Mg^2+^ replacement were significantly reduced (interaction time*treatment F(2,24) = 8.84, **P < 0.01, repeated measures ANOVA). (C) Fluorescent calcium responses to three bouts of electrical stimulation in a single neurite under control conditions (left panel), following addition of EDTA (3 mM) to the perfusate (centre panel) and after wash with re-establishment of the extracellular Ca^2+^ concentration (right panel). (D) Pooled data for 14 neurites comparing repeated electrical stimulation (grey bars, control) with the addition of EDTA (3 mM) to the extracellular solution (filled bars, EDTA). Calcium responses in the presence of EDTA (3 mM) were significantly reduced (interaction time*treatment F(2,24) = 6.45, **P < 0.01, repeated measures ANOVA).

To explore the contribution of extracellular calcium to intracellular calcium transients seen in response to electrical stimulation, the concentration of extracellular calcium was reduced either by calcium replacement or chelation. For 7 neurites (NGF), equimolar replacement of extracellular Ca^2+^ with Mg^2+^ reduced the electrically-evoked peak calcium signal from 0.32±0.06 to 0.05±0.02 (Fig 2B; n = 7, F(2,24) = 8.84, p = 0.001, interaction (time x treatment), repeated measurements ANOVA; representative example see Fig 2A). This effect was reversed upon reestablishment of the extracellular Ca^2+^ concentration at 2 mM. In a further set of 7 neurites (NGF) the extracellular Ca^2+^ concentration was reduced by addition of EDTA (3 mM). This would be expected to reduce the concentration of free Ca^2+^ to a nominal value of 0.07 mM (ref: http://www.stanford.edu/~cpatton/webmaxcS.htm). Under these conditions, the peak increase in electrically-evoked Ca^2+^ was reduced from 0.24±0.06 to 0.02±0.01 (Fig 2D; n = 7, F(2,24) = 6.45, p = 0.006, interaction (time x treatment), repeated measurements ANOVA; representative example see Fig 2C).

### Intracellular calcium responses increase with number of electrical pulses

**Fig 3:**
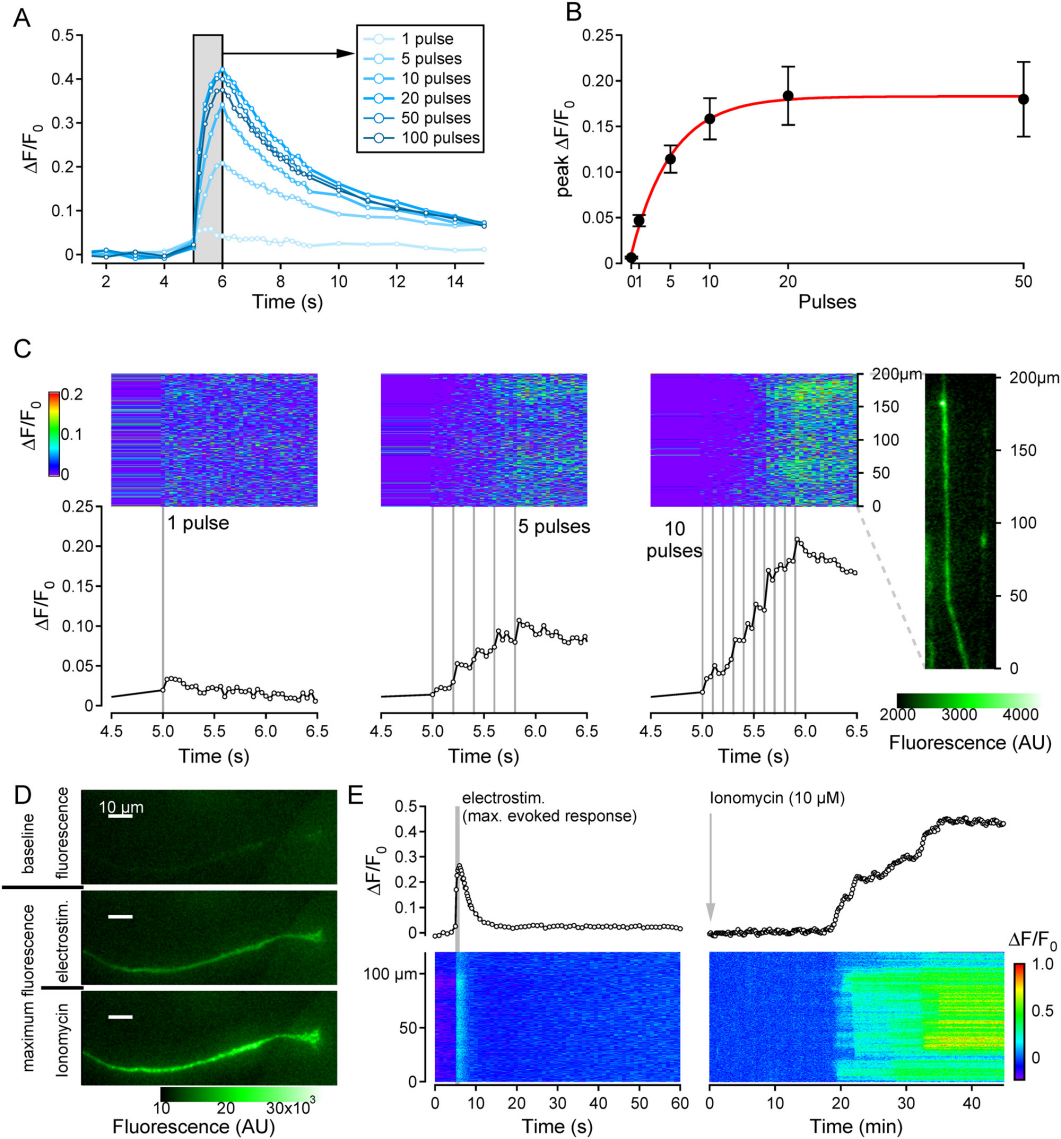
Frequency dependence of electrically-evoked calcium responses in neurites. (A) Overlay of Ca^2+^ responses in a single neurite in response to sequential stimulation with 1, 5, 10, 20, 50 and 100 pulses over 1 second (grey bar). (B) Pooled data for average peak calcium response (ΔF/F_0_, black markers) as a function of stimulus frequency with an exponential regression fit (red line). Averaged data derives from recordings from 30 neurites excepting data for 0 Pulses (n = 8), 1 Pulse (n = 29) and 50 Pulses (n = 19). Spatiotemporal profile of fluorescent calcium signals along the terminal 200μm of a single neurite (rightmost inset) in response to stimulation with 1 current pulse (left panels), 5 pulses/s (centre panels) and 10 pulses/s (right panels). Fluorescent images were acquired at 25 Hz. The upper (blue) panels show fluorescence intensity coded as color along the length of the neurite (ordinate) and as a function of time (abscissa). Lower panels show the average fluorescence signal (ΔF/F_0_, open markers) determined from all pixels along the neurite ROI as a function of time. Individual electrical stimuli (1ms, 40mA) are shown as vertical grey lines. (C) (D) Fluorescence images of a neurite before (top), during electrical stimulation (centre) and during application of ionomycin (10μm) (bottom). (E) Calcium responses for the neurite in panel D as a function of time (upper) and spatiotemporally (lower) in response to electrical stimulation (20Hz, left) and ionomycin (10μM, right).

The use of calcium signals in neurites as an index for monitoring electrically-evoked action potential number was determined by quantifying the effect of stimulus frequency on the amplitude of calcium transients. Calcium signals were determined in 30 neurites in response to 1 s bouts of electrical stimuli comprising either 1, 5, 10, 20, 50 or 100 pulses. An example of the resulting electrically evoked changes in calcium signal (ΔF/F_0_) is shown for a single neurite in Fig 3A. To improve temporal resolution, fluorescent images during and for 3 s after the stimulation period were acquired at 25 Hz (Fig 3C). The peak of the calcium signal following electrical stimulation increased with stimulus frequency up to approximately 20 Hz above which the peak calcium transient reached a maximum. Using an exponential fit, the half-maximum response amplitude corresponded to 3.02 Hz with the maximum increase in ΔF/F_0_ reaching 0.18±0.004 (Fig 3B). Interestingly, an increase in ΔF/F_0_ is clearly discernible in response to a single electrical stimulus, both in the individual neurite examples in Fig 3A and 3C as well as in the data averaged across all neurites (Fig 3B, 0.05±0.01, n = 30, p<0.01 baseline vs. 1 pulse, independent samples t-test). The ability to resolve changes in ΔF/F_0_ in response to individual electrical stimuli is also evident during stimulation at 5 Hz and 10 Hz (Fig 3C) in which distinct increases of ΔF/F_0_ coincide with individual electrical pulses (vertical grey lines, Fig 3C).

The saturation of the maximum electrically evoked ΔF/F_0_ at stimulus frequencies above 20 Hz (Fig 3B) prompted an examination of whether this was due to saturation of the calcium indicator. In 3 neurites, maximum electrically evoked ΔF/F_0_ signals were compared directly with calcium increases resulting from exposure to ionomycin (10 μM). In these neurites, average ΔF/F_0_ after ionomycin was 0.80±0.46, a value 1.5-fold higher that the peak transient response to electrical stimulation (0.53±0.15; Fig 3D and 3E), suggesting that an inability to follow higher stimulus frequencies limits the electrically-evoked calcium response amplitude (Fig 3B).

### Higher incidence of conduction block by TTX in neurites from GDNF- over NGF-cultured somata

**Fig 4:**
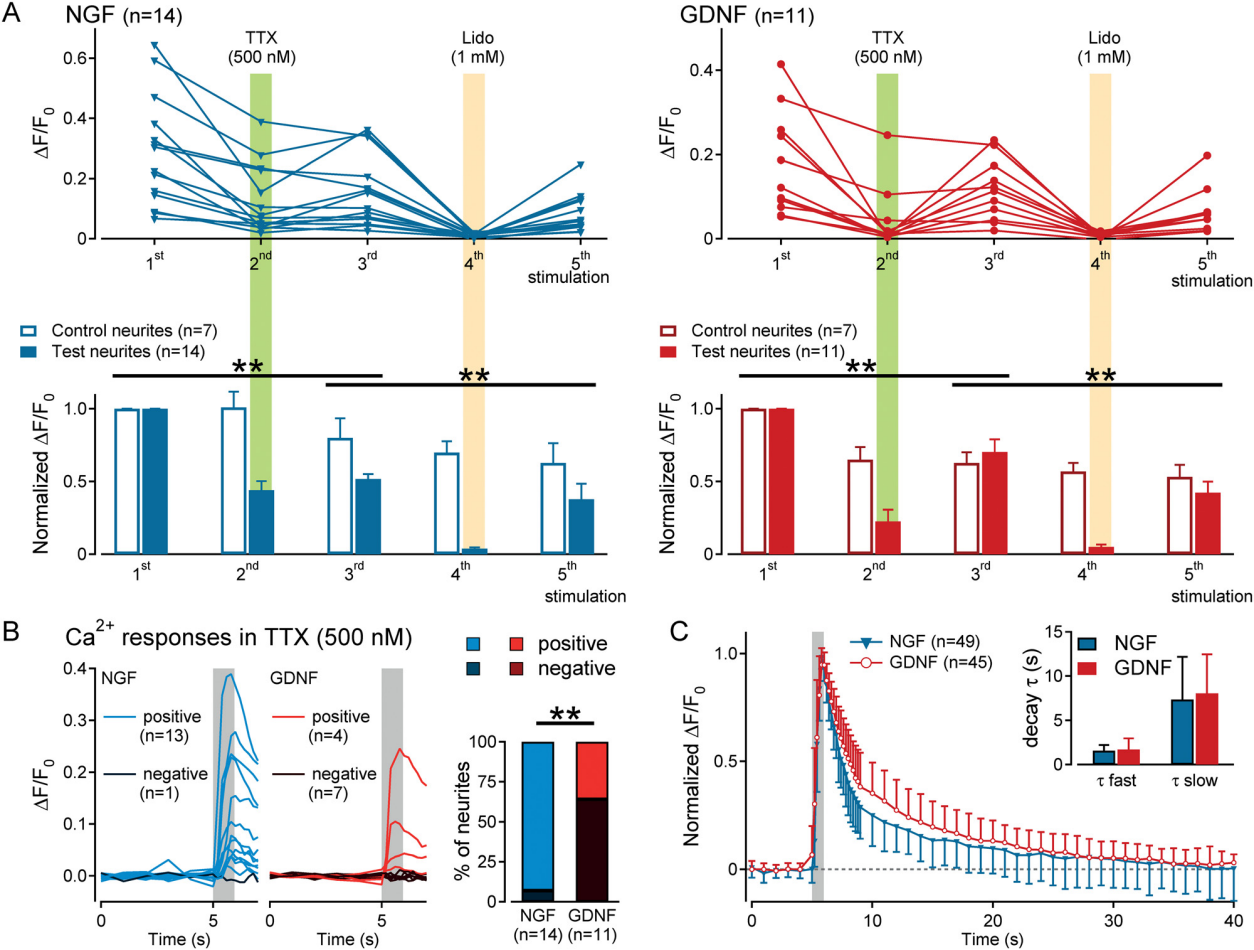
Effect of sodium channel (NaV) blockade on electrically-evoked calcium responses in NGF or GDNF neurites. (A) Calcium responses to 5 sequential bouts of electrical stimulation shown as raw data for individual neurites (upper, individual response joined by lines) and as normalized averages (lower panels) for 14 test neurites (filled blue markers in upper panel and filled blue bars lower panel) and 7 control neurites (open blue bars in lower panel) from NGF cultured somata (left panels) and 11 test neurites (filled red markers in upper panel and filled red bars lower panel) and 7 control neurites (open red bars in lower panel) from GDNF cultured somata (right panels). Tetrodotoxin (TTX, 500nM, green shading) was applied to the test neurites (shaded bars in lower panels) during the second stimulus and similarly, for the test group of neurites lidocaine (Lido, 1mM, orange shading) was applied during the fourth stimulus period. (B) Individual calcium responses to electrical stimulation (grey shading) in NGF (left panel) and GDNF (centre panel) neurites in the presence of TTX (500nM). Applying the criterion for a positive calcium response (see methods) conduction in one of 14 NGF neurites (7%) was blocked by TTX (500nM) whereas 7 out of 11 GDNF neurites (64%) were blocked by TTX (right panel). (C) Averaged normalized calcium response to control electrical stimulation (grey shading) for 94 neurites (49 NGF, 45 GDNF) illustrating the bi-exponential decay of the calcium signal. Determined for each individual response, the average fast and slow time constants of decay did not differ between NGF or GDNF neurites (inset).

The contribution of different NaV isoforms to conduction in neurites was examined using tetrodotoxin (TTX) to block TTX-s NaV isoforms (NaV1.1–1.4, NaV1.6–1.7). Exposure to TTX (500nM) in the lateral compartment reduced the electrically-evoked peak calcium response ΔF/F_0_ in both NGF and GDNF neurites (Fig 4A; NGF: n = 14, lower left, F(2,38) = 15.73, p<0.001; GDNF: n = 11, lower right, F(2,32) = 6.97, p = 0.003; interaction (time x treatment), repeated measurements ANOVA). However, TTX (500nM) in the lateral compartment resulted in complete block of electrically-evoked calcium responses in 7/11 (64%) recordings from GDNF neurites but only 1/14 (7%) NGF neurites (Fig 4B, left). NGF neurites are thus more readily able to conduct action potentials using TTX-r NaV isoforms than GDNF neurites (n = 25, p = 0.003, Pearson's chi-squared test, Fig 4B, right). In all neurites, both GDNF and NGF, calcium signals in response to electrical stimulation were abolished by lidocaine (1 mM) in the lateral compartment (Fig 4A; NGF: n = 14, lower left, F(2,38) = 5.39, p = 0.009; GDNF: n = 11, bottom right, F(2,32) = 16.27, p<0.001; interaction (time x treatment), repeated measurements ANOVA). Blockade of electrically-evoked calcium responses by lidocaine was reversed upon washout confirming that the neurites were still vital.

The decay kinetics of electrically-evoked calcium signals were quantified using double exponential fit to normalized fluorescence (Fig 4C). Across all 94 neurites, neither the fast time constant of decay (NGF neurites: n = 49, 1.55±0.67 cf. GDNF neurites: n = 45, 1.7±1.26; p = 0.466, unpaired Student’s t-test) nor the slow time constant (NGF neurites: n = 25, 7.37±4.8 cf. GDNF neurites: n = 30, 8.07±4.4; p = 0.572, unpaired Student’s t-test) differed between NGF and GDNF neurites.

## Discussion

The results establish the utility of calcium imaging for indirectly examining action potential conduction in cultured neurites of porcine DRG neurons. Calcium signals evoked electrically in porcine DRG neurons residing in the central compartment of a Campenot chamber were detected as calcium transients in neurites in the lateral compartment. Electrically-evoked calcium signals arose secondary to action potentials conducted along the neurites. The peak amplitude of electrically-evoked calcium signals was limited by the peak discharge frequency, estimated to be around 10 to 20 Hz in neurites of porcine DRG neurons at room temperature. In NGF neurites, TTX-r NaV isoforms were alone sufficient to propagate action potentials in approximately 90% of neurites, whereas TTX-r propagated calcium signals were evident in only 40% of GDNF neurites. This functional difference suggests that either NGF-responsive neurons are intrinsically disposed to elevated expression of TTX-r NaV isoforms or NGF promotes surface expression of TTX-r NaV isoforms in outgrowing neurites.

### Neurotrophic factor dependent regulation of NaV isoforms

In rodents, approximately 75% of DRG neurons are dependent upon NGF for survival during development [38]. Postnatally, approximately half reduce expression of the high-affinity trkA receptor, lose responsiveness to NGF and upregulate the tyrosine kinase Ret signaling receptor for the GDNF neurotrophic factors [27]. In the adult, these two neuronal population appear largely non-overlapping comprising either peptidergic DRG neurons expressing trkA, and the neuropeptides Substance P and CGRP, or Ret expressing neurons devoid of neuropeptides and binding the isolectin IB4 [39]. Differences in expression of the TTX-r isoforms NaV1.8 and NaV1.9 between trkA-expressing and IB4-positive DRG neurons have been reported [40]. Nav1.9 is expressed at higher levels in individual IB4-positive DRG neurons, the majority of which are C-nociceptors [41]. TrkA-expressing DRG neurons are also nociceptors and amongst A-neurons trkA expression correlated positively with NaV1.8 but this was not apparent in C-neurons [42]. An association between the trkA receptor for NGF and Nav1.8 is nevertheless consistent with the observation here that TTX-r NaVs are more able than GDNF neurites to conduct action potential along neurites from NGF-dependent DRG neurons. A parsimonious interpretation for this difference would imply that culturing the DRG neurons with either NGF or GDNF drives neurite outgrowth from two distinct neuronal subpopulations expressing different levels of NaV1.8. In pig however, this is the first study examining functional differences between sub-populations of neurons selected according to their sensitivity to NGF and GDNF. A second possibility could be that NGF and GDNF regulate different transcriptomes leading to upregulation or differential targeting of NaV1.8 to neurites by NGF [43,44,45].

### Propagated calcium signals in sensory nerve terminals

Propagated calcium signals have been examined previously in individual sensory nerve terminals in an *ex vivo* preparation of the rat cornea [46,47] as well as in neurites from mouse DRG neurons *in vitro* [48]. These studies and our results indicate that in sensory neurites and axons calcium signals evoked by electrical (Fig 2 and [46]), mechanical [48,49] or chemical (e.g. capsaicin¸ [48]) stimuli require an influx of extracellular calcium. Propagated calcium signals are in turn dependent upon NaV-mediated action potentials (Fig 4A and [48]). Combined recordings of voltage and intracellular calcium from individual DRG neurons indicate that action potential number correlates with the peak of the calcium transient [49]. Our results support this observation in neurites and further indicate that at higher calcium signal acquisition rates (25 Hz) individual action potential synchronous calcium transients, can be resolved (Fig 3C). The maximum amplitude of calcium transients observed here occurred in response to stimuli at approximately 10 to 20 Hz (Fig 3A and 3B). A range of 10-20Hz is consistent with direct electrophysiological recordings from the terminals of the outgrowing neurites of cultured mouse DRG neurons in which depolarizing current evoked firing at approximately 10 Hz (their Fig 7, [50]). Similarly, in corneal nerve terminals, peak calcium responses were observed at stimulus rates around 20 Hz [46]. The maximum calcium signal observed in our neurites does not reflect saturation of the calcium indicator as evidenced by the ability to evoke higher calcium responses with ionomycin (10 μM; Fig 3D and 3E). Therefore, the lack of further increase in calcium signals at stimulus rates of 50 and 100 Hz probably reflects intermittent failure of conduction along the neurite.

Recordings from the somata of rat DRG neurons using cytosolic [51,52,54,55] as well as luminal calcium dyes [53] all indicate that calcium-induced calcium release and store-operated calcium entry [51,56,57] both contribute to calcium signaling. Interestingly, while the magnitude of CICR differs amongst DRG neurons, being prominent in large and medium diameter neurons but essentially absent in small diameter, IB4 and capsaicin positive neurons [51] store-operated calcium entry was most prominent in small diameter DRG neurons [51]. This differential contribution to calcium signals in different sub-populations of DRG neurons suggest that NGF and GDNF dependent DRG neurons may also differ in their calcium handling, but future experiments will be need to explore this possibility. Although our data provide little insight into the role of calcium-induced calcium release Gover et al (2007) examined the contribution of ER and mitochondrial calcium stores to electrically-evoked calcium signals in sensory nerve terminals of the rat cornea. Using pharmacological tools they concluded that the clearance of cytosolic calcium following electrical stimulation was dominated by the plasmlemmal Ca^2+^-ATPase (PMCA) with little evidence for intracellular calcium sequestration and release [47].

### Role of TTX-s and TTX-r NaV isoforms in neuronal calcium signaling

TTX-r sodium currents recorded in isolated DRG neurons and attributed to NaV1.8 are thought to be functionally significant in maximizing activation of high voltage gated calcium channels [58]. In the axons of primary afferent sensory neurons calcium signals are thought to serve two important roles, driving transmitter release at central terminals and enabling neuropeptide release in the axonal reflex flare at peripheral nerve terminals. In vitro findings in sensory nerve terminals in the cornea [46] as well as in outgrowing neurites from murine [48] and porcine (Fig 4) DRG neurons both indicate that propagated calcium signals are largest when TTX-s NaV isoforms are available and substantially reduced by TTX, i.e. calcium signals are smaller when mediated by TTX-r NaV isoforms. For central terminals this is consistent with functional recordings in the dorsal horn showing that TTX-r NaV isoforms alone are not sufficient to drive pre-synaptic transmitter release from primary afferent nerve terminals in the spinal dorsal horn [21] nor the central terminals of olfactory sensory neurons in olfactory bulb [59]. In the peripheral terminals of afferent sensory neurons the contribution of TTX-r NaVs to neuropeptide release is less clear. In people lacking functional Nav1.7 skin flare responses can be evoked in response to histamine [60] and to pin prick mechanical stimuli [61], although there might also be a non-neuronally mediated component of vasodilation to both of these stimuli. Further modulatory effects of PKA- and PKC-dependent phosphorylation pathways on TTX-r NaV1.8 [62] could also potentially manifest as changes in calcium signals in axons and nerve terminals.

In summary, our results suggest that calcium transients in the neurites of dorsal root ganglia neurons can be used *in vitro* to study axonal conduction of action potentials. The higher incidence of TTX resistance in neurites from NGF- as compared to GDNF cultured neurons may reflect an enhanced expression of NaV1.8 by NGF or could reflect population differences between NGF and GDNF dependent DRG neurons in pigs.

## Supporting Information

S1 Table Raw Data table for Figs 1–4. (XLS)
